# Methods of Assessing Nailfold Capillaroscopy Compared to Video Capillaroscopy in Patients with Systemic Sclerosis—A Critical Review of the Literature

**DOI:** 10.3390/diagnostics13132204

**Published:** 2023-06-28

**Authors:** Zechen Ma, Douwe Johannes Mulder, Robert Gniadecki, Jan Willem Cohen Tervaert, Mohammed Osman

**Affiliations:** 1 Division of Rheumatology, Department of Medicine, University of Alberta, Edmonton, AB T6G 2R7, Canada; zechen5@ualberta.ca (Z.M.);; 2 Division of Vascular Medicine, Department of Internal Medicine, University Medical Centre Groningen, University of Groningen, 9700 RB Groningen, The Netherlands; 3 Division of Dermatology, Department of Medicine, University of Alberta, Edmonton, AB T6G 2R3, Canada

**Keywords:** systemic sclerosis, nailfold capillaroscopy, videocapillaroscopy, dermatoscopy

## Abstract

Introduction: Nailfolds of patients with systemic sclerosis (SSc) provide an opportunity to directly visualize microvascular remodeling in SSc. Nailfold video capillaroscopy (NVC) remains the gold standard for assessing nailfold capillaroscopy (NFC). However, access to NVC is limited by expense and expertise. This review aims to synthesize current research on other NFC devices compared to NVC. Methods: The literature search included the primary research of adult patients with SSc as defined by the 2013 ACR/EULAR criteria. Methods of assessing NFC included stereomicroscopy/wide-field microscopy, ophthalmoscopy, dermatoscopy, smartphone devices, and digital USB microscopy. Primary outcomes included both qualitative (normal vs. abnormal nailfolds, overall pattern recognition, presence/absence of giant capillaries, hemorrhages, and abnormal morphology) and quantitative (capillary density and dimension) measures. Results: The search yielded 471 studies, of which 9 were included. Five studies compared NVC to dermatoscopy, two compared it to widefield/stereomicroscopy, one to smartphone attachments, and one to USB microscopy. In dermatoscopy studies, NVC had a higher percentage of images that were interpretable (63–77% vs. 100%), classifiable (70% vs. 84%), or gradable (70% vs. 79.3%) across three studies. Dermatoscopy had a lower sensitivity (60.2% vs. 81.6%) and higher specificity (92.5% vs. 84.6%) compared to NVC. One stereomicroscopy study found a significant difference between methods in capillary density in limited cutaneous SSc, while another found correlations in all parameters between stereomicroscopy and NVC. One smartphone lens had good agreement with NVC on abnormal capillary morphology and density. USB microscopy was able to differentiate between SSc and healthy controls using mean capillary width but not by capillary density. Discussion: A dermatoscope may serve as a more portable and affordable screening tool to identify a normal “scleroderma pattern”, and images that need further corroboration by NVC. NFC parameters reported are heterogenous and the standardization of these parameters is important, especially in non-gold-standard devices.

## 1. Introduction

### Key Messages

Systemic sclerosis (SSc) is a poorly understood autoimmune connective-tissue disease characterized by fibrosis of the skin and/or internal organs, immune dysregulation, and microvasculopathy. The nailfolds of patients with SSc provide an opportunity to directly visualize microvascular remodeling in SSc. This was first visualized by Maricq and colleagues using widefield microscopy in the 1970s [1]. Since then, abnormal nailfold capillaries has been proposed as a method to distinguish primary Raynaud’s phenomenon (RP) from RP due to SSc [2].

Longitudinally, in patients with RP who meet the classification criteria for “early” SSc with no other evidence of connective tissue disease, abnormalities in images via nailfold capillaroscopy (NFC) together with autoantibodies were found to be highly predictive of developing definite disease [2,3]. Furthermore, a normal pattern on NFC combined with the absence of SSc-specific auto-antibodies rules out the presence of SSc [4]. Efforts have been combined internationally to standardize NFC [5]. Patients with SSc typically exhibit a “scleroderma-spectrum pattern” characterized by a loss of capillaries, enlarged capillary loops, capillary hemorrhage, and the disruption of capillary appearance [6]. As a result of NFC’s diagnostic value, it has been included in the 2013 American College of Rheumatology (ACR)/European League Against Rheumatism (EULAR) classification criteria for SSc [7].

**Figure 1:**
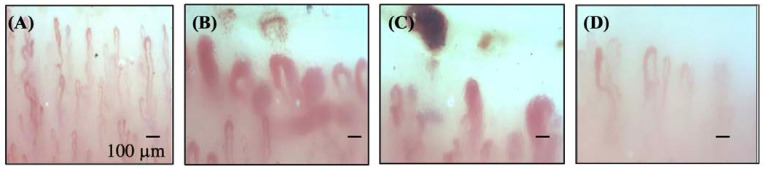
Representative NVC images from various patients. (**A**) Idiopathic Raynaud’s phenomenon without autoantibodies; (**B**) Active SSc pattern without ILD or PAH in a patient with early SSc; (**C**) Active SSc pattern in a patient with SSc and ILD, (**D**) Late SSc pattern with PAH. Abbreviations: SSc: systemic sclerosis, DM: dermatomyositis. Note the decreased capillary density in (**C**,**D**); and the giant capillaries (>50 mm in apical diameter) in (**B**,**C**) associated with increased microhemorrhages. All images were captured using a DS Medica nailfold video capillaroscopy device at a 200× magnification.

In addition to its diagnostic value, emerging evidence suggests that NFC parameters can be used in prognostication and disease monitoring in SSc. These parameters include the presence of giant capillaries, microhemorrhages, decrease in capillary density, and presence of avascular areas or disorganized angiogenesis [8] (as illustrated in Figure 1). Microhemorrhages and giant capillaries (>50 mm in apical diameter) have been associated with the active phase of disease, with a stronger correlation seen with microhemorrhages than with giant capillaries [9]. An increase in capillary apical width and decrease in capillary density were also found in patients with SSc-pulmonary arterial hypertension (PAH) [10]—although this may not be only present in PAH associated with SSc. A decreased capillary density on NFC represents the proliferative microvasculopathy that is well described in patients with PAH. Intriguingly, capillary density is inversely associated with presence of SSc-interstitial lung disease (ILD), the longitudinal progression of SSc-ILD, and the presence and severity of SSc-PAH by right-heart catheterization [11,12]. Additionally, some have found capillary loss in ‘late’ SSC to be associated with the increased risk of digital skin ulcers [13,14]. This may stem from decreased capillary loss and a subsequent chronic hypoxic state systemically—particularly in acral tissues such as the digits [8]. Further to that, “late” NFC changes representing disorganized angiogenesis as a consequence of a chronic hypoxic state may also be detectable [8]. This disorganized form of angiogenesis prospectively correlates with the development of SSc-PAH [12].

**Figure 2:**
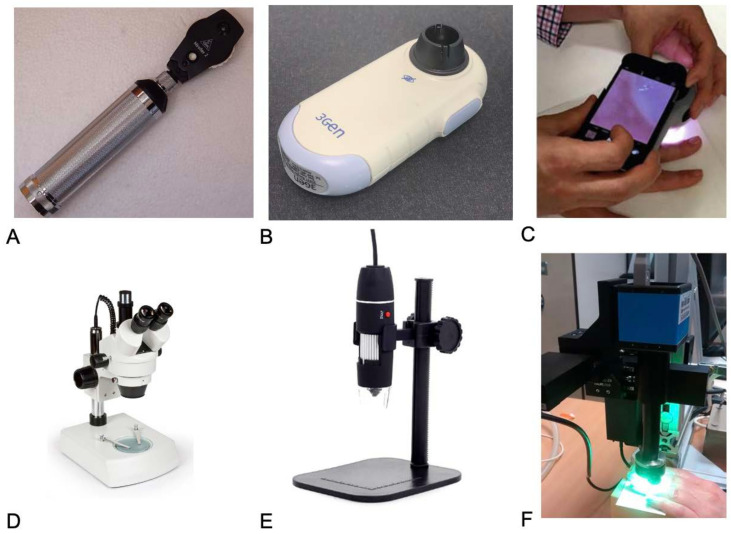
Examples of various devices used for visualizing capillaries in patients with SSc. (**A**) ophthalmoscope, (**B**) dermatoscope, (**C**) smartphone attachments, (**D**) stereomicroscope, (**E**) USB attachments, (**F**) nailfold videocapillaroscope.

Numerous methods have been described to assess nailfold capillaries including the stereomicroscope, dermatoscope, ophthalmoscope, smartphone devices, digital USB microscopes, and the current gold-standard device, the nailfold videocapillaroscope (NVC) (Figure 2, Table 1). Nailfold videocapillaroscopy combines a microscope with a digital video camera with the magnification ranging between 50× and 500×. The nailfold images are then stored and analyzed on specific parameters in each single frame separately and adjacent images can be combined to visualize the entire nailfold by software [15]. In addition, a capillary score that is comprised of features associated with SSc-related disease activity (e.g., giant/enlarged capillaries and microhemorrhages) and vascular remodeling (e.g., capillary disorganization, loss, and dropout) is generated after the examination [8]. Hence, NVC has become the accepted as the “gold-standard”. However, access to NVC is mostly restricted to centers with special interests due to expense and required expertise when assessing NFC visually. In fact, according to a survey of SSc specialists in mostly academic hospitals in the USA found that while 91% of experts assess NFC “always” or “most of the time”, 64% use a dermatoscope or ophthalmoscope for evaluation, and only 7% use NVC [16]. Despite growing interest in the utility of NFC in SSc, there are no systematic reviews that compare the different devices against the gold standard, NVC. This systematic review synthesizes evidence comparing different NFC devices to NVC in assessing NFC in patients with SSc.

## 2. Materials and Methods

The review included primary research from observational studies with a comparison group. Studies on adults (age 18 or older) with SSc were included. Systemic sclerosis was defined by the 2013 ACR/EULAR classification criteria. Methods of assessing NFC included but were not limited to stereomicroscopy/wide-field microscopy, ophthalmoscopy, dermatoscopy, smartphone devices, and digital USB microscopy [5]. Nailfold video capillaroscopy was defined as a microscope with a digital camera with a magnification that ranges between 50× to 500×. Studies that included NVC and another form of capillaroscopy assessment were included. The primary outcomes of interest included qualitative (normal vs. abnormal nailfolds, overall pattern recognition (scleroderma pattern), or quantitative (capillary density, capillaries with abnormal morphology, capillary dimension, and presence/absence of hemorrhage) outcomes captured by different devices. There were no limitations on publication date or publication type. The search was limited to the English language.

The literature search included articles that were published until 23 May 2022 in MEDLINE, EMBASE, and Web of Science. A combination of the following keywords was used including: capillaroscopy, USB, smartphone, stereomicroscopy, dermatoscopy, nailfold video capillaroscopy, ophthalmoscopy, and SSc. The search terms were developed based on equipment names used in a previously published review by Smith et al. [5]. The search strategy is included in Supplementary Materials.

Title and abstract screening (level 1), full text (level 2) screening, and data abstraction was completed by one reviewer (ZM) then confirmed by a second reviewer (MSO). Abstracted data included authors, year of publication, country of publication, sample size, eligibility criteria, NFC assessment device, NVC device, assessors, outcomes, and main results. Covidence was used to eliminate duplicates and screen records. Given the heterogeneity of outcomes reported and the results of included studies, the results were synthesized narratively.

## 3. Results

The search resulted in 138, 330, and 3 studies from MEDLINE, EMBASE, and Web of Science, respectively. After duplicates were removed, 457 titles and abstracts were screened. Fourteen abstracts underwent full-text screening. Nine studies met the final inclusion criteria. Five studies compared NVC to dermatoscopy, two compared it with widefield/stereomicroscopy, one with smartphone attachments, and one with USB microscopy.

### 3.1. Videocapillaroscopy vs. Dermatoscopy

Five of nine included studies compared dermatoscopy to NVC (Table 2) [17,18,19,20,21]. The sample size across studies ranged from 20 to 170 participants. Two included studies were from the United States, two were from the United Kingdom, and one from Turkey. The dermatoscopes used, videocapillaroscopes used, and outcomes measured were heterogenous across studies. Radic and colleagues in the United States compared two dermatoscopes including a non-contact polarised HEINE Delta 20 T dermatoscope connected to a digital camera with 10–16× magnification (D1), as well as DermLite DL3 dermatoscope with 10× lens with a DermLite connection kit for iPad mini 4 (D2) in a group of 100 patients with RP. This group developed an algorithm based on the presence or absence of scleroderma patterns (reduced capillary density, capillary enlargement, hemorrhages, and capillary morphology), and categorized images to normal, definite scleroderma, non-specific, and non-interpretable groups. According to the algorithm, non-specific and non-interpretable groups underwent further evaluation with NVC. In this group, the images considered normal or definite scleroderma by dermatoscope were consistent with NVC classifications. There was a higher percentage of non-interpretable images with dermatoscopes (37% using D1, 23% using D2), compared to non-interpretable images with NVC (0%). From the developed algorithm, 50% of dermatoscopic images in this group were non-specific or non-interpretable, which required further imaging using NVC. All scleroderma patterns by NVC were found to be either a non-specific or definite scleroderma pattern by dermatoscope [17]. Hughes et al. included 32 patients (8 controls, 3 primary RP, and 21 patients with SSc spectrum disorders) [18]. Their group used a semi-quantitative scale based on the severity of abnormalities ranging from 0 (normal) to 3 (grossly abnormal). Similarly in this group, there was a significant difference in classibility between NVC vs. dermatoscope (84% vs. 70%, *p* < 0.001). Additionally, severity scores were significantly higher in NVC (1.69, 95% CI 1.44–2.94) than in dermatoscopy 1.26 (1.04–1.49). The correlation between two techniques on classibility was 0.4 (95% CI 0.3–0.49) by the same rater, and for severity was 0.65 (95% CI 0.55–0.73) [18]. Dinsdale et al. had the largest sample size of 170 patients (99 SSc patients, and 71 controls) who underwent dermatoscopy from 10 expert observers from seven centers. Nailfolds were assessed as either ungradeable (due to the extreme severity of the abnormality or poor image quality) or gradeable. Gradeable nailfolds then were classified as normal, non-specific, early, active, or late SSc. Gradeability was 70.9% using dermatoscopy and 79.3% using NVC. With dermatoscopy, sensitivity was 60.2% and specificity was 92.5% whereas sensitivity was 81.6% and specificity was 84.6% with NVC to identify disease status of the study participant (non-SSc or SSc) [19]. Dogan et al. compared the DermLite ProGen with a digital camera and found moderate agreement (k = 0.52) between the dermatoscope and NVC in classifying patients with SSc (*n* = 39) into early-phase, late-phase, and active-phase disease groups [20]. In an abstract presented at ACR, Stever and colleagues assessed SSc patients using a DermLite dermatoscope compared to NVC (Optilia) with two trainees and two experts [21]. Of those 20 patients, the dermatoscope was able to recognize abnormal capillaroscopy in 13 of 20 patients (65%) [21]. Hence, in contrast to NVC, NFC interobserver variability may be a prominent feature.

### 3.2. Stereomicroscopy vs. Nailfold Videocapillaroscopy

Two included studies compared stereomicroscopy to NVC [22,23] (Table 3). Widlt and colleagues measured capillary density (loops/mm) using two stereomicroscopy methods (direct counting using stereozoom microscope and stereozoom equipped with DeltaPix camera) with NVC in 62 patients (41 Ssc and 21 controls). The median capillary density was found to be 4.3, 5.4, and 6.1 loops/mm in limited cutaneous SSc (lcSSC) and 4.5, 5.0, and 6.3 in diffuse cutaneous SSc (dcSSC) when measured by direct counting, stereozoom with digital camera, and with NVC, respectively. Within patients with lcSSC, the capillary density was significantly different between the three methods [22]. In Brazil, a study with a larger group of 252 patients (101 with SSc, 61 with RP associated with undifferentiated connective tissue disease, 37 primary RP, and 52 healthy controls) found that widefield stereomicroscopy under 10–25× magnification correlated with NVC in all parameters including number of capillaries (r = 0.874), enlarged capillaries (r = 0.902), giant capillaries (r = 0.882), microhemorrhages (r = 0.601), and avascular score (r = 0.814) [23]. The area-under-ROC-curve analysis showed widefield NFC and the videocapillaroscopy showed similar performances in discriminating between SSc patients and healthy controls [23].

### 3.3. Smartphone Attachments

One abstract published by an Australian group compared three iPhone microscopic lenses (Olloclip ×30 magnification, Nightstar ×60 magnification, and GoMicro ×60 magnification) compared to widefield binocular nailfold capillaroscopy (×20–80 magnification) and Capiscope NVC (×100–300 magnification) in 10 patients with SSc (Table 3) [24]. This found that the Nightstar lens was superior to other smartphone lenses with good agreement on the assessment of abnormal capillary morphology and density compared to widefield binocular NFC and NVC [24]. 

### 3.4. USB Microscopy

One pilot study recruited 20 patients with lcSSc and 20 healthy controls (Table 2). Nailfold capillaroscopy was completed using XCSOURCE TE389, a USB microscope [25]. This group was able to obtain high-quality NFC images using USB capillaroscopy but the morphology in some frames was obscured. The parameters assessed included mean capillary width and capillary density. USB microscopy was able to differentiate between patients with SSc and healthy controls using mean capillary width, with an area under the ROC of 0.81 (SE 0.120) with USB microscopy compared to that of NVC (0.81, SE 0.095). USB microscopy was unable to discriminate between SSc and healthy controls based on capillary density [25].

## 4. Discussion

This review has summarized existing evidence comparing methods of assessing NFC to the current gold standard, NVC. Most of the current evidence comparing the utility of other NFC devices to NVC focused on dermatoscopy. Of these studies, evidence is consistent that a higher proportion of images acquired by dermatoscopes were non-interpretable or non-classifiable compared to those acquired by NVC [17,18,19]. Compared to NVC, the dermatoscope was less sensitive but more specific in detecting abnormalities in classifiable images [18]. In terms of scleroderma pattern subclassifications (early, active, and late), there is moderate agreement between dermatoscopy and NVC found in a small group of patients. This may be explained by the lower magnification of dermatoscopes and the fact that they are unable to evaluate capillaries in sufficient detail compared to NVC. When comparing a stereomicroscope with NVC, one study found a significant difference in capillary density between stereomicroscopy and NVC [22]. However, a study using a larger sample found that widefield stereomicroscopy strongly correlated with all parameters related to scleroderma pattern including capillary density, enlarged capillaries, giant capillaries, and avascular areas with excellent intra–inter-observer reliability for both techniques with experienced observers [23]. The search yielded only one study comparing other techniques such as smartphone attachments and USB microscopy [24]. The limited data show that smartphones microscopy had good agreement on abnormal capillary morphology and density compared to NVC. USB microscopy was able to differentiate patients with SSc from the control group using capillary width measurements, but not based on capillary density [24].

Nailfold capillaroscopy is essential to the assessment of SSc. However, the gold-standard method, NVC, is not always available and can be limited by cost as well as its laborious process [5]. The results of this review suggest that dermatoscope and stereomicroscopy may be useful alternatives to NVC. Despite lower magnification, dermatoscope may serve as a screening tool to identify normal, “scleroderma pattern”, and images need further corroboration by NVC (either non-interpretable or non-specific). However, one may expect that a high proportion of patients will require NVC corroboration as 50% of dermatoscopy images required further NVC [17]. Additionally, dermatoscopy may have some ability to differentiate between scleroderma patterns (early, active, late) based on limited evidence. Dermatoscopes have the advantage of portability as well as being affordable [5]. These advantages are reflected in a survey of SSc experts in the USA in 2019, which indicated that 64% of clinicians use a dermatoscope or ophthalmoscope, with only 7% using NVC [16]. Additionally, training curriculums have been developed to use dermatoscopy in rheumatology fellowship programs [26]. On the other hand, stereomicroscopy showed a strong correlation in all parameters related to scleroderma pattern compared to NVC, with the ability to discriminate between SSc patients from controls by experienced observers. Though not portable, stereomicroscopy has the advantage of being able to visualize the whole nailfold microvasculature for the prompt localization of abnormalities and is the more cost-effective compared to NVC [5]. However, stereomicroscopy may not be able to capture high quality NFC images in patients with SSc with finger-flexion ankylosis. Current evidence is scarce but promising in using smartphone microscopy, and USB microscopy to assess NFC in patients with SSc, though further research is needed. Other potential tools that are similar include epiluminescence microscopes (e.g., Dino-Lite US), as these devices generate high-quality digital images compared, albeit, at a lower magnification compared to the NFC gold standards, but with a fraction of the cost [27]. However, their specificity and/or sensitivity have not been directly compared. 

On the other hand, as reflected in the articles included in this review, NFC parameters measured are heterogenous and not consistently reported. These parameters assessed by NVC have important clinical implications. For example, NVC-detected capillary density is a reliable predictor of overall disease progression, the occurrence of digital ulcers, the progression of pulmonary disease, and skin fibrosis [11,12,28]. Additionally, capillary density can be used to monitor the effects of treatment [29]. Using NVC, the inter-rater and intra-rater reliability for NVC is excellent across specific capillaroscopy parameters including capillary-loop dimensions, widened and giant capillaries, and capillary density [12]. The reliability of these specific quantitative metrics in other devices is not well studied. While one of the included studies reported excellent interobserver and intraobserver agreement for widefield microscopy to classify images by specific capillaroscopic parameters (capillary density, enlarged capillary loops, microhemorrhages, giant capillary loops, avascular score) [23], the reliability of dermatoscopes to study these metrics may be limited by its magnification and are not yet well described [30,31].

This is the first review to synthesize current evidence comparing NVC to other methods for assessing NFC to our knowledge. This review has captured studies from the USA and Europe, which may have captured differences in practice patterns [16,32]. Additionally, this review included all publication types, which also included conference abstracts that added to available evidence.

The limitations of this review included its language limitation as well as limitations on the search strategy’s inclusion of different equipment terminology. Another limitation is the inclusion of only patients with SSc, when there is evidence to suggest NFC changes in other connective-tissue disorders [33,34]. The results from this review may not extrapolate to other settings as it is not clear in included studies how much training/expertise evaluators had when assessing nailfolds with different methods. In 2020, Smith and colleagues proposed the standardization of the assessment of NFC by NVC in SSc [5]. The emergence of portable and low-cost imaging techniques makes NFC assessment in SSc more accessible. More research is needed to evaluate performance characteristics using standardized parameters on NVC in larger studies.

## Figures and Tables

**Table 1:** Nailfold capillaroscopy devices.

	Magnification	Cost
Ophthalmoscope	Up to 15×	Low ($200–1000)
Dermatoscope	Up to 10×	Low to medium ($1000 and up)
Smartphone attachments	Up to 20×	Variable ($50 and up)
USB devices	Variable, up to 300×	Low to medium ($20 and up)
Stereomicroscope	Variable, up to 200×	Medium to high ($1000–5000)
Videocapillaroscope	50–500×	High ($10,000 and up)

**Table 2:** Dermatoscopy vs. Videocapillaroscopy (*n* = 5).

Last Author (Country, Publication Year)	Participants Number of Nailbeds Assessed	Dermatoscope (Assessor)	Nailfold Videocapillaroscopy (Assessor)	Outcomes	Main Findings
Radic (USA, 2020) [17]	100 consecutive RP patients 2–5th fingers of both hands.	Handheld non-contact polarised dermatoscope connected to digital camera with 10–16× magnification assessed by experienced assessor DermLite DL3 dermatoscope and connector kit for iPad mini 4 by one assessor and reviewed by experienced reviewers	Videocapillaroscope with 200× magnification.	EULAR group on microcirculation in rheumatic diseases into scleroderma patterns or non-scleroderma patterns using an algorithm depending on presence and absence of (qualitatively reduced capillaries, enlargement, hemorrhages, and morphology) then categorize to normal, scleroderma (Definite), non-specific, vs. non-interpretable	Higher non-interpretable (37 using D1, 23 using D2 vs. 0 with NVC). 50% of dermatoscopic images were non-specific vs. non-interpretable and required NVC using an algorithm. Dermatoscopic normal were consistent with NVC. Scleroderma pattern on dermatoscopy were corroborated by scleroderma pattern on NVC. All scleroderma by NVC were found to be non-specific or scleroderma by dermatoscopy.
Hughes (UK, 2015) [18]	32 patients: 8 controls, 3 primary RP, and 21 SSc-spectrum disorders. 10 nailbeds per patient	Dermatoscope not-specified 48 raters from 12 countries	NVC, not specified	Semi-quantitative scale on severity of abnormality: 0 (normal), 1 (mildly abnormal), 2 (definitely abnormal), 3 (grossly abnormal), and unclassifiable	84% vs. 70% (*p* < 0.001) classifiable on NVC vs. dermatoscope. Severity score higher in NVC than dermatoscopy 1.69 (1.44–2.94) vs. 1.26 (1.04–1.49) Correlation between NVC and dermatoscope by same rater was 0.4 (CI 0.3–0.49) for classibility. Between techniques for severity was 0.65 (CI 0.55–0.73).
Dinsdale (UK, 2018) [19]	170 participants: 99 SSc, 71 controls 1376 nailfolds	Handheld dermatoscopy (×10 magnification) by 10 expert observers from seven centers	NVC with 300× magnification	Graded based on: - Ungradeable: extreme severity of capillaroscopic abnormality or image quality - Gradeable: normal, non-specific, early, active, or late	Gradeability was 70.9% in dermatoscopy and 79.3% in NVC. Normal (DS vs. NVC): 47.2% vs. 29.1 Early: 9.2 vs. 9.6% Active: 11.0% vs. 15.6% Late: 7.1% vs. 9.5% Non-specific: 25.5% vs. 36.2% Of non-specific, majority of patients had Ssc (65.9% in DS vs. 56.6% with NVC). Sensitivity was 60.2% and specificity was 92.5% (273/295 of non-SSc images correctly identified) by dermatoscope. Sensitivity was 81.6% and specificity was 84.6% in NVC group.
Stever (USA, 2017) [21]	20 SSc patients Eight digits assessed 153 images	DermLite by two trainees and two experts	Nailfold video capillaroscopy (NVC Optilia) by two trainees and two experts	Unclear	Dermatoscope able to recognize abnormal capillaroscopy in 13 of 20 patients (65% of the time)
Dogan (Turkey, 2013) [20]	39 SSc patients	DermLite ProGen (3Gen, San Huan Capistropano) with Sony Cybershot DSC-W220	Videocap Net DS Medica (200× magnification)	Groups based on dermatoscopy: Capillary dilatation Giant capillaries and avascular aeras Disrupted vascular configuration Both giant capillaries and dilatation Matched early phase (group 1, 2), late phase (3), and active phase (4)	Cohen’s kappa agreement between groups is k = 0.52.

**Table 3:** Nailfold capillary assessment using different devices compared to nailfold videocapillaroscopy.

Last Author (Country, Publication Year)	Participants Number of Nailbeds Assessed	NFC Device Used (Assessor)	Nailfold Videocapillaroscopy (Assessor)	Outcomes	Main Findings
Wildt (Sweden, 2012) [22]	40 patients with SSc	Direct counting (DC) of capillaries along 3 mm in center of nailfold using stereo-zoom microscope (Olympus SZ-Pt, Japan) at 20× magnification Stereozoom microscope with DeltaPix camera (DP, 200, DeltaPix, Denmakr) (IA) By one assessor	Using KK Technologies, Honiton, Devon, UK at 300× magnification By one assessor	Capillary density	DC, IA and CNVC, in lcSSc patients was median (range) 4.3 (2.3–6.7), 5.4 (3.0–7.3) and 6.1 (2.9–8.6) loops/mm, and in the dcSSc patients was 4.5 (2.3–5.0), 5.0 (3.0–7.3) and 6.3 (2.1–6.9) loops/mm. In controls, the median (range) was 7.0 (5.7–9.7), 7.0 (3.7–10.3) and 6.9 (5.0–10.0) loops/mm. Significance of the difference in capillary density between SSc and controls was *p* < 0.001 (DC), <0.001 (IA) and *p* = 0.01 (CNVC) for lcSSc patients, and *p* < 0.001 (DC), *p* = 0.01 (IA) and *p* = 0.05 (CNVC) for dcSSc patients. Capillary density in lcSSc patients assessed by DC was lower than that obtained with IA (*p* < 0.001), and lower than that obtained with NVC (*p* < 0.001). IA capillary density was lower than CNVC (*p* < 0.05). In dcSSc patients, DC significantly different between IA and NVC. No difference between IA and NVC. No difference in controls between methods.
Sekiyama (Brazil, 2013) [23]	252 patients (101 with SSc, 61 with RP associated with undifferentiated CTD, 37 primary RP, and 52 controls)	Widefield NFC using stereomicroscope (SZ40, Olympus) under 10–25× magnification (no video capture)	Videocapillaroscopy 200× magnification connected to Videocap 8.14 DS Medica.	Number of capillaries/mm, number of enlarged capillaries, number of giant capillaries, number of microhemorrhages. Placed into three patterns: normal pattern, nonspecific microangiopathy, and scleroderma pattern	Significant correlation (*p* < 0.000) between widefield NFC and NVC in all parameters: capillaries/mm r = 0.874, enlarged capillaries r = 0.902, giant capillaries r = 0.882, microhemorrhages r = 0.601, and avascular score = 0.814. To discriminate between SSc and non-SSc: ROC for number of capillaries/mm showed AUC of 0.906 (*p* < 0.001) using widefield NFC vs. 0.935 with NVC. For avascular score, it showed an AUC 0.955 using NFC and 0.947 using NVC. ROC analysis for number of capillaires/mm showed an AUC of 0.858 with widefield and 0.894 using NVC. Reliability: for three different patterns (normal, nonspecific, and scleroderma pattern), there was almost perfect interobserver and intraobserver agreement for widefield (k = 1, *p* < 0.001) and NVC (k = 0.917, *p* < 0.001).
Patterson (Australia, 2020) [24]	10 patients with SSc	Olloclip (×30 magnification) Nightstar (×60) GoMicro (×60)	Widefield binocular NFC (×20–80) and Capiscope NVC (×100–300)	Not reported	Nightstar ×60 lens superior to others. Good agreement in abnormal capillary morphology and density with traditional methods. Less defined capillary morphology using smartphone attachments
Berks (UK, 2021) [25]	20 patients with lcSSc and 20 controls	XCSOURCE TE389 device from Amazon	NVC	Capillary density, average width of capillaries	High-quality images can be generated using USB microscopy. USB microscope mean width from controls was 15.0 lm (95% CIs 12.5, 17.4), and in patients with SSc 31.2 lm (95% CI 23.7, 38.6), ROC AZ: 0.81 (SE 0.120). Mean width on NVC in controls was 12.1 lm (95% CI 11.4, 12.9) vs. SSc 19.0 lm (95% CI 16.1, 21.8). ROC AZ: 0.81 (SE 0.095). Mean density using USB microscope controls was 4.68/mm (95% CI 2.75, 6.61) vs. SSc 3.78/mm (95% CI 2.71, 4.68) ROC AZ = 0.48 (SE 0.16). NVC mean density in controls was 6.43/mm (95% CI 5.19, 7.67) vs. SSc 3.99/mm (95% CI 3.12, 4.87), ROCAZ = 0.70 (SE 0.10).

## Data Availability

The authors confirm that the data supporting the findings are available within the article and its Supplementary Materials.

## Supplementary Materials

The following supporting information can be downloaded at: https://www.mdpi.com/article/10.3390/diagnostics13132204/s1, File S1: Search Strategy.
