# Supplementary material for: Methods of Assessing Nailfold Capillaroscopy Compared to Video Capillaroscopy in Patients with Systemic Sclerosis—A Critical Review of the Literature
Source: Diagnostics (Basel). 2023 Jun 28;13(13):2204. doi: 10.3390/diagnostics13132204 (PMC10341321; doi:10.3390/diagnostics13132204)
Supplement: Supplementary file 1 [file diagnostics-13-02204-s001.zip › diagnostics-2406700-supplementary.pdf]

Supplementary Materials S1: Search strategy

MEDLINE:
("Scleroderma, Systemic"[Mesh] OR "systemic sclerosis" OR scleroderma) AND ("nailfold videocapillaroscop*" OR nailfold video#capillaroscop*" OR "NVC") AND ("dermatoscop*"OR "opthalmoscop*" OR "stereomicroscop*" OR "smartphone" OR "USB" OR "capillaroscop*")

EMBASE:
("Scleroderma, Systemic"[Mesh] OR "systemic sclerosis" OR scleroderma) AND ("nailfold videocapillaroscop*" OR nailfold video#capillaroscop*" OR "NVC") AND ("dermatoscop*"OR "opthalmoscop*" OR "stereomicroscop*" OR "smartphone" OR "USB" OR "capillaroscop*")
